# Biofilm in Chronic Sinusitis with Nasal Polyps: Pilot study

**DOI:** 10.1016/S1808-8694(15)30538-3

**Published:** 2015-10-19

**Authors:** Thiago Freire Pinto Bezerra, Francini Grecco de Melo Pádua, Allex Itar Ogawa, Eloisa Maria Mello Santiago Gebrim, Paulo Hilário Nascimento Saldiva, Richard Louis Voegels

**Affiliations:** Otorhinolaryngologist; Doctorate degree. Assistant physician at the Otorhinolaryngology Division, Clinical Hospital, Medical School, Sao Paulo University - USP; Medical resident at the Otorhinolaryngology Division, Clinical Hospital, Medical School, USP; Doctorate degree. Assistant physician of the Radiology Division, Clinical Hospital, Medical School, USP; Senior associate professor, full professor at the Pathology Department, Medical School, Sao Paulo University - USP; Senior associate professor, associate professor of the Clinical Otorhinolaryngology Discipline, Medical School, USP. Director of the Rhinology Sector, Clinical Hospital, Medical School, USP

**Keywords:** biofilms, nasal polyps, sinusitis

## Abstract

Chronic rhinosinusitis' pathogenesis is not completely established and there are some explanations for this disease, such as osteitis, superantigens, fungal-mediated hypersensitivity and, more recently, biofilms. There are no reports in Portuguese about biofilms in chronic rhinosinusitis.

**Aim:**

To reproduce a method for visualization of biofilms in patients with chronic rhinosinusitis and nasal polyps.

**Patients and Methods:**

Samples of ethmoid bulla of nine patients with chronic rhinosinusitis with nasal polyps without response to clinical treatment who underwent surgery were analyzed with scanning electron microscopy to evidence bacterial biofilms.

**Study design:**

A contemporary cross-sectional cohort study

**Results:**

In 55.56% (5/9) of the patients we observed biofilms by seeing three-dimensional structures, spherical structures surrounded by an amorphous matrix and water-channels.

**Conclusion:**

We reproduced a method for visualization of bacterial biofilms by scanning electron microscopy and evidenced its presence in chronic rhinosinusitis with nasal polyps.

## INTRODUCTION

Chronic rhinosinusitis is one of the most common complaints in North-American medical visits, and one of the main reasons for antibiotic prescriptions and leave of work. About 135 in 1,000 persons – 31 million people - are affected yearly in the US; the total yearly cost is estimated at 6 billion US dollars.^1^, ^2^, ^3^

A classification of rhinosinusitis according to time separates them into acute cases (lasting up to 12 weeks) and chronic (lasting over 12 weeks). Chronic rhinosinusitis includes a group with no nasal polyps and another with nasal polyps.^4^, ^5^, ^6^ Histopathological findings are difference in chronic rhinosinusitis with and without nasal polyps; the former contains an eosinophil infiltrate, while the latter has a neutrophil infiltrate, reflecting a different pathophysiology.^4^ Voegels and Padua have also suggested other differences in the inflammatory response, such as a significant decrease in the amount of interleukins in chronic rhinosinusitis with nasal polyps that progressed to a good postoperative outcome.^7^

Several theories have been raised to explain the pathophysiology of chronic rhinosinusitis. It is currently thought that chronic rhinosinusitis is an immunological inflammatory disease caused simultaneously or singly by several factors, such as: immune conditions, intrinsic upper airway factors, Staphylococcus aureus superantigens, fungal colonization that induces and maintains eosinophilic inflammation, metabolic disorders such as aspirin hypersensitivity, and persistent insult by biofilms and/or osteitis.^4^

Biofilm consists of gathered microorganism cells anchored irreversibly to a live or inert surface, encased in a self-produced extracellular polymer matrix consisting mostly of polysaccharides, which comprises over 90% of the biofilm mass.^8^, ^9^, ^10^, ^11^ This conditions makes biofilms highly resistant to changes in pH, temperature, and antibiotic action, which possibly explains persistent chronic infections that resist clinical therapy, such as chronic rhinosinusitis with nasal polyps.^8^, ^9^, ^10^, ^11^

Post (2001) carried out the first study to assess the presence of biofilms in otorhinolaryngology, in which this author applied scanning electron microscopy (SEM) to identify biofilms in ventilation tubes, associating these structures with otitis media in an animal model.^12^ Biofilms have also been demonstrated in cholesteatomas, chronic tonsillitis, adenoids of patients with chronic sinusitis, and infections associated with biomaterials such as voice prostheses.^13^, ^14^, ^15^

Perloff and Palmer conducted several studies that confirmed the presence of biofilms on the mucosa of patients with chronic rhinosinusitis;^16^, ^17^, ^18^ these biofilms could explain why such patients improved after a course of antibiotics and relapsed after medication was ceased.^19^ Other studies applying transmission electron microscopy and confocal laser microscopy with fluorescence in situ hybridization have demonstrated the presence of bacteria inside biofilms.^19^, ^20^, ^21^

No papers have been published in Portuguese about bacterial biofilms in chronic rhinosinusitis. The purpose of this study was to reproduce a method for demonstrating bacterial biofilms in chronic rhinosinusitis; this is one of the aims of a wider set of studies being conducted at the Rhinology Division of the Otorhinolaryngology Discipline at our institution. Concluding this step was essential to apply this tool for further studies.

## AIM

The purpose of this study was to identify bacterial biofilms on the mucosa of patients with chronic rhinosinusitis and nasal polyps.

## PATIENTS AND METHODS

### Patients

This was a contemporary cohort cross-sectional study carried out from February to May 2008. The report comprises the first nine chronic rhinosinusitis patients with nasal polyps that did not respond to medical therapy, and that are part of a prospective cohort study that is being undertaken at a tertiary level hospital. All patients agreed to sign the free informed consent form that was authorized by the Institutional Review Board of the hospital (number 0669/07).

Chronic rhinosinusitis with polyps was defined based on clinical and endoscopic criteria, as follows: a clinical history containing two or more of the following symptoms lasting over 12 weeks, one of the symptoms being any of the first two of nasal block or congestion, anterior nasal discharge or pos-nasal drip, facial pain or sense of pressure, and decreased or absent olfaction;5 endoscopy revealing bilateral nasal polyps.^5^, ^6^

Absence of response to medical therapy was defined as a time period over three months with no improvement after using topical nasal corticosteroids with or without oral antibiotics, leukotriene antagonists and/or inhibitors, isotonic nasal saline solutions, and/or systemic corticosteroids. Patients were aged 18 years or above.

Patients with an optical microscopy histological analysis of nasal polyps showing predominantly non-eosinophilic cell infiltrates, with secondary causes of chronic rhinosinusitis (fungus ball, invasive fungal disease, granulomatous diseases, vasculitis, mucoceles alone, nasosinusal malignant and benign tumors, congenital anomalies (such as primary ciliary dyskinesia, cystic fibrosis, craniofacial congenital anomalies, oroantral fistulae, and primary and secondary immune deficiencies) were excluded.

We collected tissue samples of the ethmoidal bulla of these patients when carrying out functional endoscopic sinus surgery (FESS) from February to April 2008.

### Method

Tissue specimens measuring about 1.0 × 1.0 cm were harvested during surgery done by one of the medical residents of our unit, and fixed for 2 hours in 2% glutaraldehyde and buffered with 0.15 M phosphate (pH 7.2) at room temperature. Each specimen was washed three times in a lavage solution containing NaCl (1.2 g), sucrose (14.6 g), and distilled water (200 ml), and post-fixed for 1 hour in 1% OsO4. Samples were then dehydrated in a series of increasing concentrations of ethyl alcohol (70% to 100%), critical point dried in CO_2_, and placed on mounts for SEM. Platinum coating was done for observation with a Quanta 600 FEG scanning electron microscope; the acceleration voltage was 10kV. Photomicrographs were captured in TIFF format at 1,000 to 12,000 magnifications. An experienced pathologist in ultra-structures (P.H.N.S) searched for images of bacterial biofilms in photomicrographs by looking for tree-dimensional structures containing spherical structures enveloped by an amorphous matrix and the water channels connecting these spherical structures. Microbiological cultures were not performed.

## RESULTS

SEM was carried out to analyze the samples of nine patients; biofilms were found in 5 of the 9 patients (55.56%), based on published criteria.^9^, ^10^, ^12^, ^13^, ^16^, ^17^, 19, 20, ^22^ The sample comprised seven men and two women with a mean age of 37 years ±11 years (22-60 years). The mean Lund-Mackay staging was 14.4 ±1.2 (12-16), and the mean Meltzer classification was 2.8 ±0.4(2-3) to the right and 3±0.5(3-4) to the left (Table 1). The three-dimensional structure, spherical structures enveloped by an amorphous matrix, and water channels connecting these spherical structures were visualized (Figure 1a, Figure 1b, Figure 2, Figure 3, Figure 4, Figure 5). Cilia were demonstrated in one case only; the epithelium of the remaining patients was devoid of cilia, showing squamous metaplasia and absence of goblet cells (Fig. 6).

**Table 1:** List of patients with data on the presence of biofilms, sex, age, tomographic (Lund-Mackay) and endoscopic (Meltzer) classifications.

Biofilm	Sex	Age	Lund-Mackay	Meltzer D/E
+	M	26	15	3/4
+	M	21	14	3/3
–	M	41	12	3/3
+	M	60	15	3/4
–	F	36	13	3/3
–	M	41	16	3/3
+	M	35	15	2/3
–	M	36	15	3/3
+	F	37	15	2/4

+ Present, - Absent, M - Male, F - Female, D - Right nasal fossa, E - Left nasal fossa

**Figure 1a:**
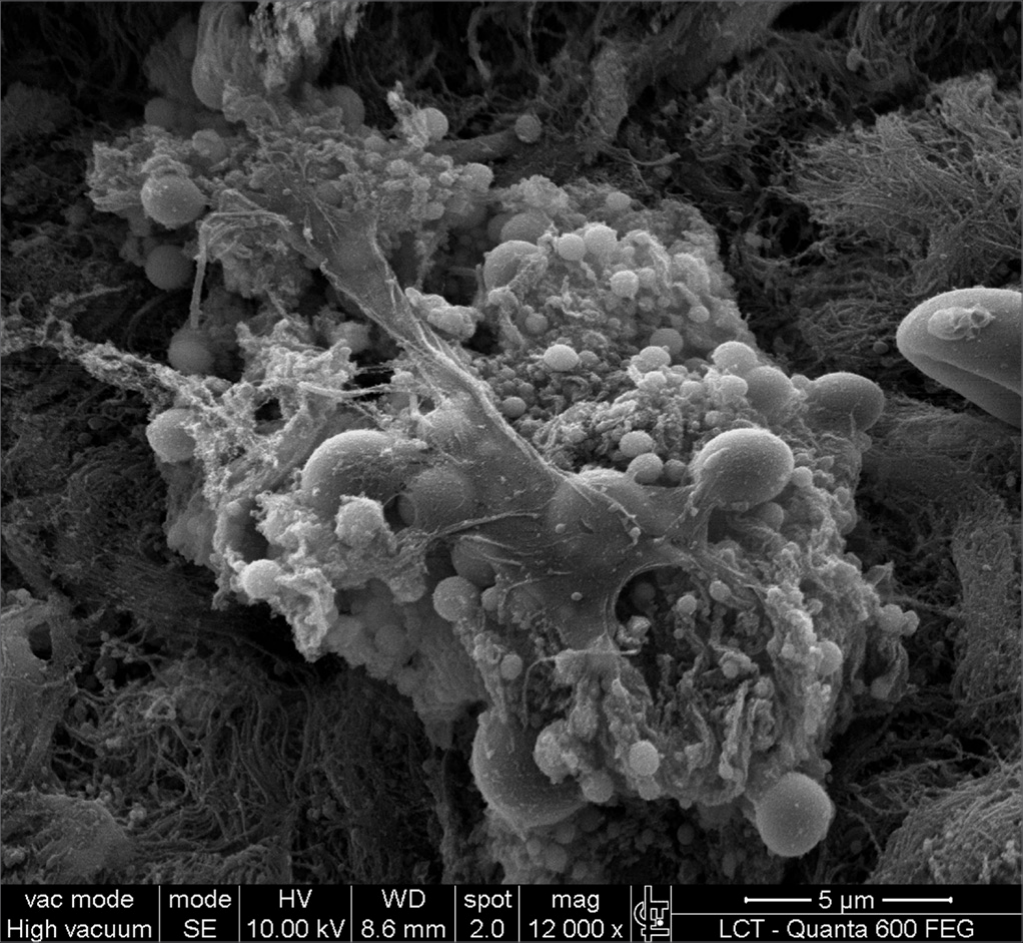
Bacterial biofilm (12.000x)

**Figure 1b:**
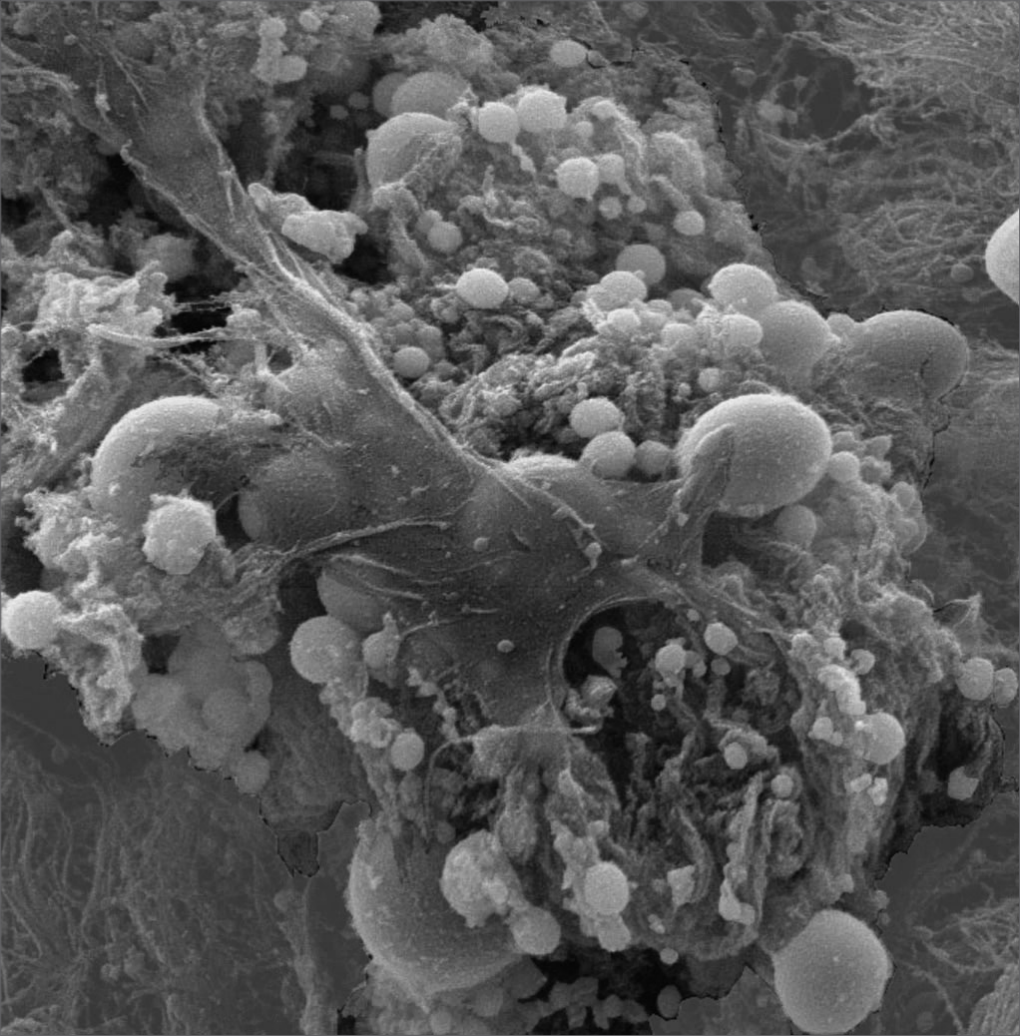
Bacterial biofilm - illustration

**Figure 2:**
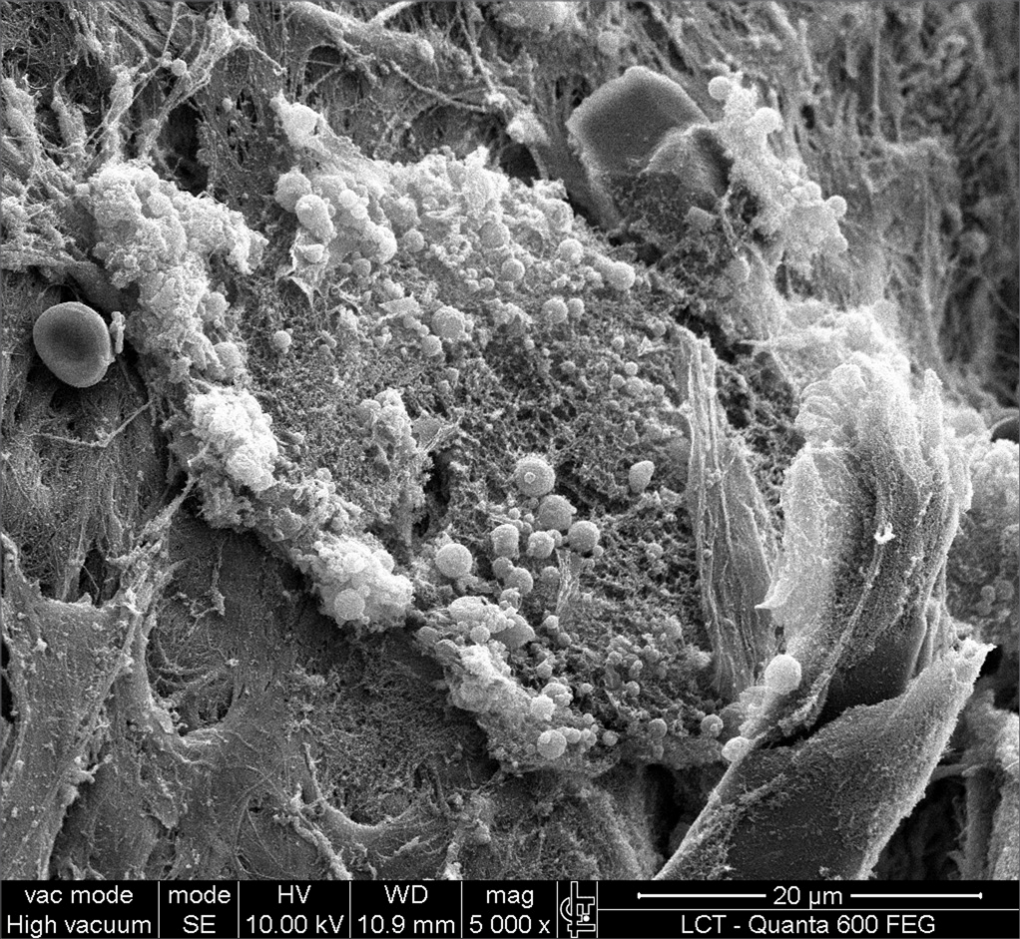
Bacterial biofilm (magnified 5.000x)

**Figure 3:**
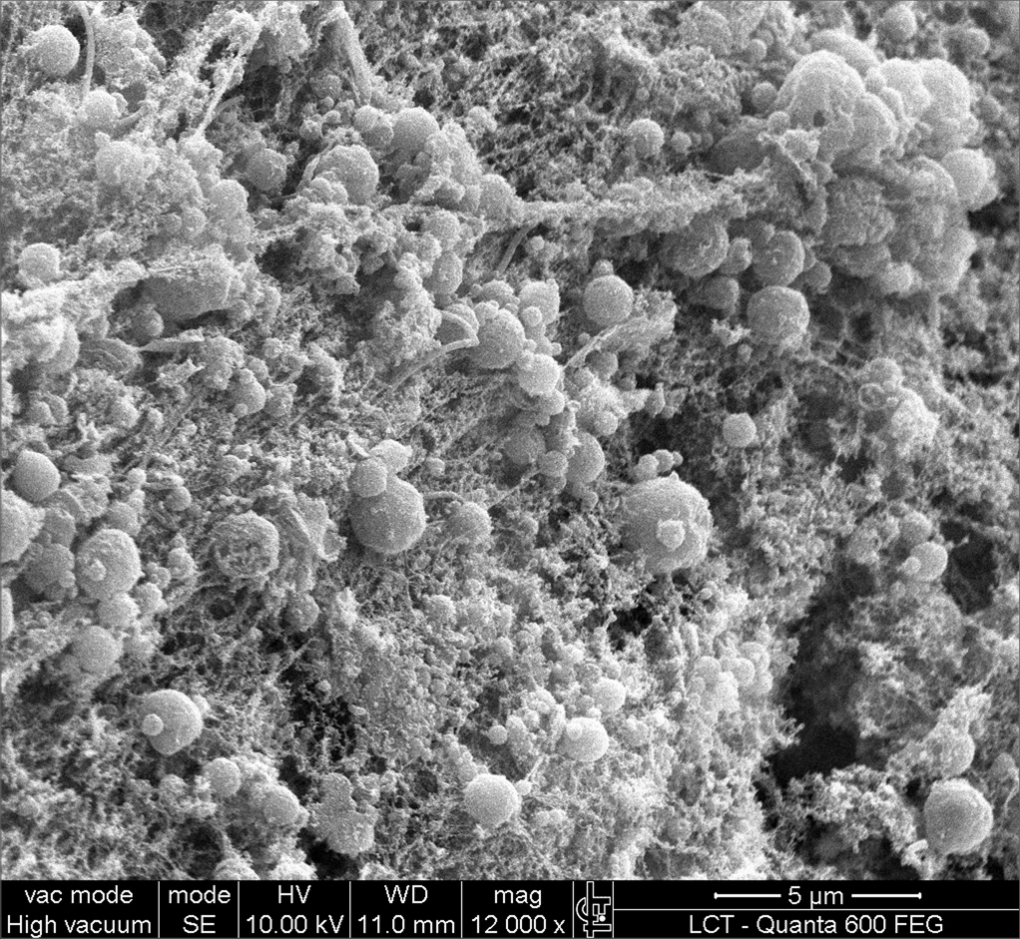
Bacterial biofilm (magnified 12.000x)

**Figure 4:**
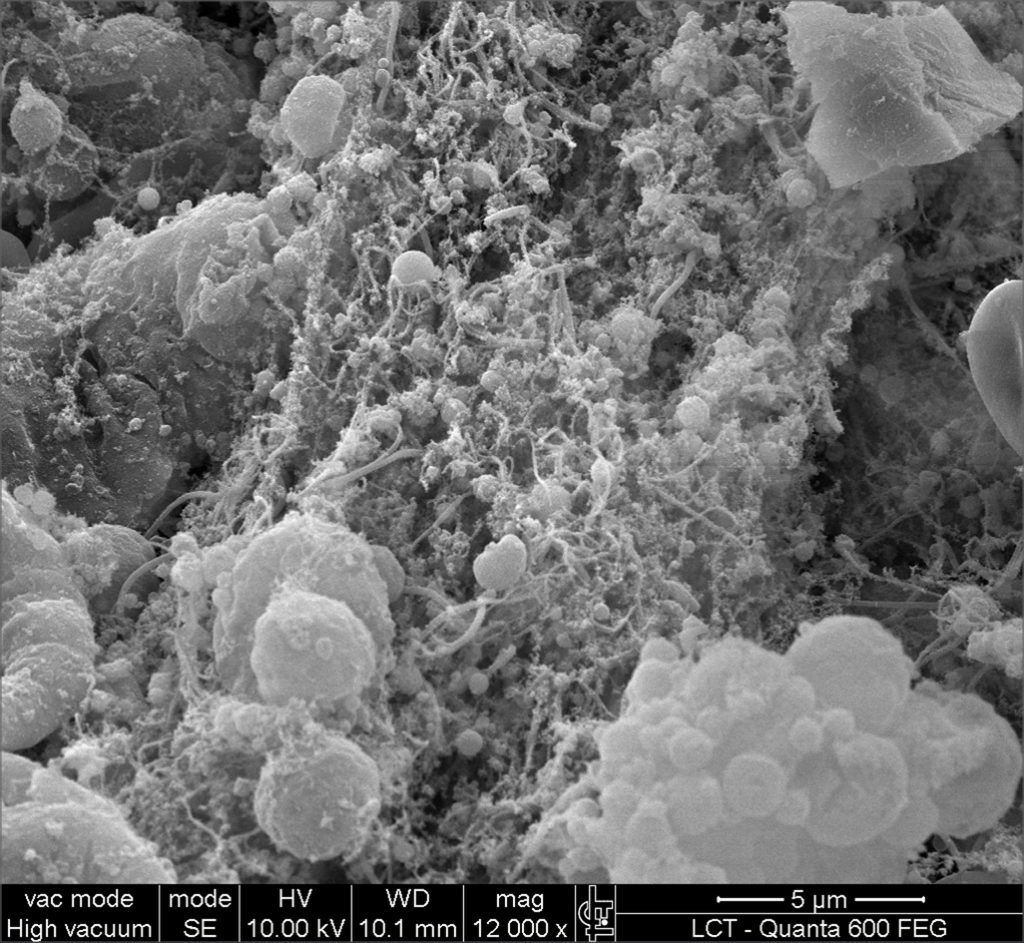
Bacterial biofilm (12.000x)

**Figure 5:**
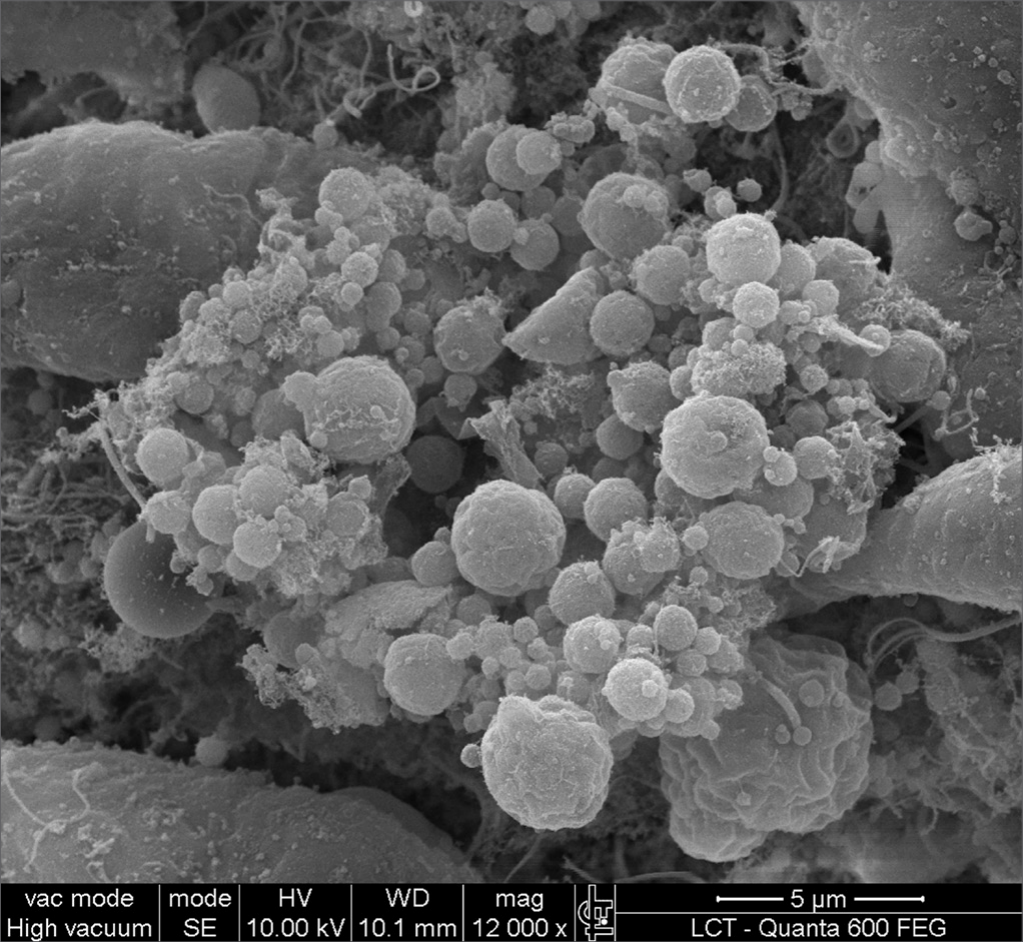
Bacterial biofilm (12.000x)

**Figure 6:**
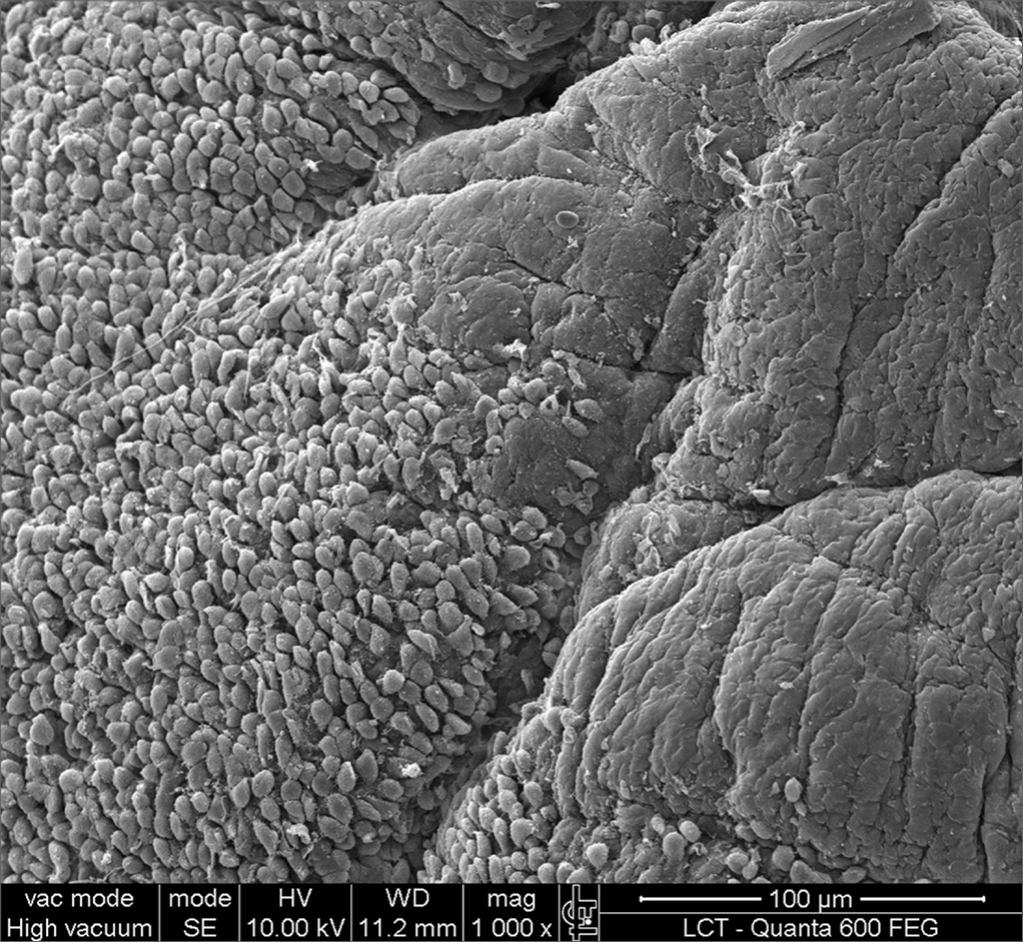
Non-ciliated cylindrical epithelium next to the squamous metaplasia

## DISCUSSION

Patients with chronic rhinosinusitis presented complaints that persisted in time, often regressing with antibiotics and relapsing after their cessation. Biofilms may explain why antibiotics are unable to eliminate this chronically established bacterial population. Bacteria in biofilms are enveloped by a glycopolysaccharide matrix and may grow coordinately after a certain cell density is reached, by inducing several signaling molecular, which is known as quorum sensing.^19^ This is a complex group of bacteria anchored to a surface, clustered in a tower and mushroom-shaped syncytium.^17^

Biofilms are less susceptible to antibiotics, grow slowly and generate planktonic bacteria intermittently (which are more susceptible to host defenses and antibiotics). These free-living bacteria appear to be responsible for the symptoms of infection.^19^

The importance given to human diseases is recent also in our specialty.^13^ Although in the past, microbiologists have looked at bacteria in their planktonic form, medical recalcitrance to antibiotics has increased interest in the behavior of bacteria when they colonize surfaces and produce biofilms.^22^ Recent papers of the North-American Centers for Disease Control and Prevention (CDC) have estimated that at least 65% of all chronic bacterial infections in humans involve biofilms.^8^, ^23^ Relevant organisms in otorhinolaryngological diseases have been shown to form biofilms, such as Pseudomonas aeruginosa, Haemophilus influenzae, Streptococcus pneumoniae and Staphylococcus aureus.^13^

Besides chronic rhinosinusitis, bacterial biofilms have been demonstrated in other disease such as suppurative otitis media, prostatitis, osteomyelitis, bacterial endocarditis, cystic fibrosis, pneumonia, and severe tonsillitis.^13^

Difficulties in demonstrating biofilms in cultures of patients with chronic rhinosinusitis may be explained by the presence of a gene - in P. aeruginosa it is the pvrR gene - that becomes active in response to specific environmental conditions; in common culture media, the bacteria does not form biofilms and is susceptible to antibiotics.^24^ Other studies have shown that bacterial culture findings correlate poorly with the presence of biofilms and the types of bacteria within.^21^

Biofilms demonstrated by SEM in the present study was confirmed with images that are similar to those published in previous studies.^17^, ^18^ Biofilms were identified in five (55.56%) of nine subjects in this pilot study. This highlights the importance of reevaluating the current treatments of chronic rhinosinusitis, because antibiotics have already been shown to be ineffective against biofilms. We point out that only mucosa of the ethmoidal bulla was harvested; possibly samples from other facial sinuses might have yielded different results. Surgical ventilation, mechanical disruption of biofilms and detergents may become therapeutic choices. Surgery may be effective because it causes the infected cavity to be ventilated, thus increasing the oxygen tension in the ambience around biofilms.^17^ Biofilms are not always detected, even in studies of guinea pigs with chronic rhinosinusitis. In one of these studies, the number of guinea pigs with biofilms detected by confocal microscopy was smaller than those detected with SEM (48% × 86%). It is thought that confocal microscopy is more precise than SEM because it specifically detects bacteria; in these papers, however, markers for other agents present in biofilms, such as fungi, were not used.^25^

A possible limitation of SEM is that preparation of the specimen involves dehydration and may cause protein cross-links that give rise to artifacts similar to biofilms. Dehydration reduces the size of biofilms and minimally distorts its architecture; it remains, however, easily recognizable, as demonstrated by comparison with cryofixation. It is possible that biofilms may be extracted during preparation or that they may not be seen by being too small.^20^

Recent studies have correlated the presence of biofilms with a worse prognosis in patients with chronic rhinosinusitis.^20^, ^26^, ^27^ Other studies have sought alternative methods for removing biofilms, such as using children's shampoo.^28^ Surgical failure may be attributed to biofilms when these are not eradicated. Biofilm persistence in the folds of edematous and chronically inflamed mucosa in which cilia are absent may lead to rapid reinfection.^29^

Other factors are also relevant in chronic rhinosinusitis with nasal polyps, since polyps were not seen in nearly half of such patients in this study. The bacterial superantigen, osteitis, and fungus-mediated hypersensitivity also contribute significantly to the pathogenesis of chronic rhinosinusitis; these factors were not investigated in this pilot study, although they modify the progression of chronic rhinosinusitis with polyps.^4^, ^30^ It is not known how much each of these factors contributes to the genesis or maintenance of chronic rhinosinusitis. Similarly, the presence of biofilms in tissue samples of patients with chronic rhinosinusitis and polyps is a fact; it remains unclear whether biofilms in such cases are the cause or consequence of persistent infection.

In this study we reproduced a biofilm detection method, which may provide more tools for studies seeking solutions for chronic rhinosinusitis. This is the first paper on biofilms published in Portuguese. The progression of patients with chronic rhinosinusitis and polyps is consistent with the current knowledge about biofilms, where there are relapses after using antibiotics and improvement after the biofilm mass is removed; additional studies, however, are needed to define whether biofilms are the cause or consequence in chronic rhinosinusitis with polyps.

## CONCLUSION

This study evidences the presence of biofilms in chronic rhinosinusitis patients with nasal polyps, showing their 3-dimensional structure, spherical structures surrounded by an amorphous matrix and its water channels.
